# Treatment initiation and completion among head and neck squamous cell carcinoma patients in Tanzania

**DOI:** 10.1186/s13104-024-07045-7

**Published:** 2024-12-23

**Authors:** Mary Jue Xu, Sumaiya Haddadi, Beatrice Paul Mushi, Li Zhang, Godfrey Sama, Sarah Kutika Nyagabona, Dianna Ng, Sikudhani Muya, Atuganile Edward Malango, Enica Richard, Patrick Ha, Sue S. Yom, Willybroad Massawe, Elia J. Mmbaga, Katherine Van Loon, Aslam Nkya

**Affiliations:** 1 Department of Otolaryngology-Head and Neck Surgery, University of California San Francisco, San Francisco, United States of America; 2 National Clinician Scholars Program, University of California San Francisco, San Francisco, United States of America; 3 Muhimbili University of Health and Allied Sciences, Dar es Salaam, Tanzania; 4 Department Epidemiology and Biostatistics, Department of Medicine, University of California San Francisco, San Francisco, United States of America; 5 The College of Surgeons of East, Central and Southern Africa (COSECSA), Arusha, Tanzania; 6 The Branch for Global Surgical Care, University of British Columbia, Vancouver, Canada; 7 Department of Internal Medicine, Division of Oncology, Muhimbili National Hospital, Dar es Salaam, Tanzania; 8 Department of Pathology, Memorial Sloan Kettering Cancer Center, New York, United States of America; 9 Department of Radiation Oncology, Ocean Road Cancer Institute, Dar es Salaam, Tanzania; 10 Department of Anatomic Pathology, Muhimbili National Hospital, Dar es Salaam, Tanzania; 11 Department of Otorhinolaryngology, Muhimbili University of Health and Allied Sciences, Dar es Salaam, Tanzania; 12 Department of Radiation Oncology, University of California San Francisco, San Francisco, United States of America; 13 Department of Otorhinolaryngology, Muhimbili National Hospital, Dar es Salaam, Tanzania; 14 Department of Epidemiology, Muhimbili University of Health and Allied Sciences, Dar es Salaam, Tanzania; 15 Department of Medicine, Division of Hematology/Oncology, University of California San Francisco, San Francisco, United States of America

**Keywords:** Head and neck cancer, Tanzania, Africa, Treatment incompletion

## Abstract

**Objective:**

Few studies characterizing clinical outcomes of head and neck cancer (HNC) patients in sub-Saharan Africa report the proportion of patients who initiate and complete treatment, information integral to contextualizing survival outcomes. This retrospective cohort study describes HNC patients who presented to Muhimbili National Hospital and Ocean Road Cancer Institute in 2018, the highest-volume oncology tertiary referral centers in Tanzania. Logistic regression was applied to assess predictors of treatment initiation and completion.

**Results:**

Among the 176 head and neck squamous cell carcinoma (HNSCC) patients, 34% (59) had no treatment documented, 34%(59) had documentation of treatment initiation but not completion, and 33%(58) had documentation of treatment completion based on the modalities started. Univariate logistic regression showed that late-stage disease was associated with increased odds of initiating treatment (OR 8.24, 95% CI 2.05–33.11, *p* = 0.003) and trends toward completing treatment (OR 7.41, 95% CI 0.90–60.99, *p* = 0.063). At last visit, 36.9%(65) were alive with a median follow up of 5.6 months (IQR 1.64—12.5 months). A large proportion of HNC patients who presented to MNH and ORCI did not initiate or complete treatment. These metrics are critical to contextualize care outcomes of HNC patients in resource-constrained health systems and develop interventions.

**Supplementary Information:**

The online version contains supplementary material available at 10.1186/s13104-024-07045-7.

## Background

Literature on head and neck cancer (HNC) outcomes in Africa is both limited and does not consistently report details on treatment initiation and completion. In a meta-analysis on HNC from 2002 to 2022, data only represented 10 of the 54 African countries, despite almost 20% of the global population residing on the continent [1, 2]. While the meta-analyses reported a 54.4% 5-year overall survival, few studies discussed details such as the proportion of patients who initiated and completed treatment, details critical to contextualizing clinical outcomes [1]. The few studies that did report these metrics noted 15–40% of HNC patients never received any treatment after a diagnoses and that up to 30% of patients who received chemotherapy or radiation did not complete treatment [3–9]. More data is needed to supplement this limited literature.

Muhimbili National Hospital (MNH) and Ocean Road Cancer Institute (ORCI) are the highest-volume oncology tertiary referral centers in Tanzania. At MNH, the distribution of HNCs was previously assessed in a study of 113 patients over a seven-month period starting in 2012; findings from the study noted a mean age at diagnosis of 51 years, lower than described in Western populations [10–12]. Head and neck squamous cell carcinoma (HNSCC) was the most common pathology (74%), and the most common anatomic sites of malignancy were sinonasal and larynx [10]. The study provided a broad description of patient demographics and tumor presentation; however, treatment and clinical outcomes were not included.

To better describe treatment adherence and to complement prior studies on HNC in sub-Saharan Africa, this study aims to assess factors associated with treatment initiation and completion among HNC patients who present to MNH and ORCI in Tanzania.

## Methods

### Study setting

In Tanzania, cancer care is mainly based at a few tertiary referral centers [13]. MNH and ORCI are the largest national oncology referral centers providing cancer care to over 5,000 new patients a year. MNH and its associated teaching institution Muhimbili University of Health and Allied Sciences (MUHAS) treat the highest number of cancer patients surgically in the country. ORCI is a national center for radiation and medical oncology care with two external beam radiation machines and two brachytherapy machines.

### Data collection

All pathology-confirmed HNC patients presenting to MNH and ORCI in 2018 were included. Patients with thyroid cancer were excluded. Medical records were identified by their diagnoses both in paper charts and through the electronic pathology records.

Data abstracted included clinical presentation, physical examination, imaging reports, AJCC 7th tumor stage (clinical staging when pathologic staging not available), pathology, and clinical outcomes. For patients treated at both MNH and ORCI; the medical records from two institutions were paired using a single study identifier. Missing data were coded as unknown. De-identified data were entered into a REDCap™ database [14]. 

Treatment initiation was defined as the patient having documentation of the start of any type of treatment. Treatment completion was defined as a patient completing the planned treatment for each modality started. For surgery, this would involve completion of surgery. For radiation, the radiation course was categorized as incomplete or complete based on documentation by the provider. For chemotherapy, the number of chemotherapy cycles planned and completed were documented. It is possible that care received at other centers may not have been documented in the medical records. Vital status was based on last clinical encounter.

### Statistical analysis

Median and interquartile ranges (IQR) described non-parametric data including age and duration of symptoms. Chi-squared test with exclusion of unknown data compared proportions of patient demographics, diagnostic evaluation, and disease characteristics between institutions as well as treatment groups (no treatment, treatment initiation, treatment completion). Wilcoxon rank sum and Kruskal-Wallis one-way ANOVA tests compared median age and duration of symptoms for non-parametric data.

Univariate logistic analyses were performed, selecting predictors hypothesized to increase the probability of initiation and completion of treatment in the case of increasing proximity to the hospitals (Coastal zone is the zone in which MNH and ORCI are located), later-stage disease, and institution (ORCI). Other predictors included increasing age and head and neck subsites (oral cavity compared other subsites). Predictors which reached a significance threshold of *p* < 0.10 on univariate analysis were included in a multivariate logistic regression model.

Missingness of data for variables was assessed relative to outcome. Specifically, among the predictor variables, overall cancer stage was missing at the highest proportion of over 40% (Table 1). As a result, missingness at random of stage was compared to the outcomes of treatment initiation and treatment completion. Missing stage compared to documented stage was significantly associated with treatment initiation but not treatment completion using a chi-squared test. Given that missing data for stage could not be considered random, the logistic regressions were performed excluding patients whose predictors variables were missing to minimize data biases.

**Table 1:** Head and neck squamous cell carcinoma patient demographics, evaluation, and clinical status by treatment initiation and completion

		All Head and Cancer Squamous Cell Carcinoma Patients		Group 1: No Treatment		Group 2: Treatment Initiated but Not Completed		Group 3: Treatment Completed		*p*
		**n**	%	**n**	%	**n**	%	**n**	%	
Total		176		59		59		58		
Age (years)	Median*	55.9		60.0		54.6		53.8		0.174
	IQR	46.2 - 66.8		49.7 - 70.1		44.7–64.4		46.2 - 64.7		
Sex	Male	118	67.0%	42	71.2%	42	71.2%	34	58.6%	0.385
	Female	56	31.8%	17	28.8%	17	28.8%	22	37.9%	
	Unknown	2	1.1%	0	0.0%	0	0.0%	2	3.4%	
Zone	Coastal	85	48.3%	33	55.9%	27	45.8%	25	43.1%	0.248
	Northern	44	25.0%	8	13.6%	19	32.2%	17	29.3%	
	Central	14	8.0%	8	13.6%	2	3.4%	4	6.9%	
	Southern Highlands	17	9.7%	6	10.2%	4	6.8%	7	12.1%	
	Zanzibar	8	4.5%	3	5.1%	3	5.1%	2	3.4%	
	Lake	3	1.7%	0	0.0%	1	1.7%	2	3.4%	
	Western	3	1.7%	1	1.7%	2	3.4%	0	0.0%	
	Unknown	2	1.1%	0	0.0%	1	1.7%	1	1.7%	
Tobacco Use	Yes, Any	69	39.2%	22	37.3%	28	47.5%	19	32.8%	0.323
	No	58	33.0%	17	28.8%	18	30.5%	23	39.7%	
	Unknown	49	27.8%	20	33.9%	13	22.0%	16	27.6%	
Alcohol Use	Yes, Any	73	41.5%	21	35.6%	27	45.8%	25	43.1%	0.374
	No	61	34.7%	24	40.7%	17	28.8%	20	34.5%	
	Unknown	42	23.9%	14	23.7%	15	25.4%	13	22.4%	
HIV	Positive	12	6.8%	2	3.4%	5	8.5%	5	8.6%	0.768
	Negative	36	20.5%	8	13.6%	17	28.8%	11	19.0%	
	Unknown	128	72.7%	49	83.1%	37	62.7%	42	72.4%	
Symptom Duration (weeks)	Median **	24		16		24		30		0.042
	IQR	12–52		10–32		12–51		16–52		
	Range	2–416		4–416		4–208		4–156		
Imaging	Head and Neck	150	85.2%	45	76.3%	55	93.2%	50	86.2%	0.033
	Chest	148	84.1%	45	76.3%	53	89.8%	50	86.2%	0.114
	Abdominal US	48	27.3%	9	15.3%	21	35.6%	18	31.0%	0.034
Biopsy	Any method	167	94.9%***	56	94.9%	57	96.6%	54	93.1%	0.690
Head and Neck Subsites	Oral Cavity	56	31.8%	16	27.1%	17	28.8%	23	39.7%	0.396
	Larynx	33	18.8%	13	22.0%	11	18.6%	9	15.5%	
	Hypopharynx	25	14.2%	10	16.9%	11	18.6%	4	6.9%	
	Nasopharynx	19	10.8%	5	8.5%	10	16.9%	4	6.9%	
	Oropharynx	14	8.0%	6	10.2%	3	5.1%	5	8.6%	
	Paranasal Sinus	10	5.7%	4	6.8%	3	5.1%	3	5.2%	
	Salivary Gland	7	4.0%	1	1.7%	1	1.7%	5	8.6%	
	Neck	2	4.0%	1	1.7%	0	0.0%	1	1.7%	
	Other	7	1.7%	1	1.7%	3	5.1%	3	5.2%	
	Unknown	3	1.1%	2	3.4%	0	0.0%	1	1.7%	
Overall Stage	I	6	3.4%	3	5.1%	1	1.7%	2	3.4%	0.053
	II	15	8.5%	12	20.3%	2	3.4%	1	1.7%	
	III	21	11.9%	5	8.5%	7	11.9%	9	15.5%	
	IV	49	27.8%	19	32.2%	14	23.7%	16	27.6%	
	Unknown	85	48.3%	20	33.9%	35	59.3%	30	51.7%	

* All patients *n* = 174, Group 1 *n* = 59, Group 2 *n* = 58, Group 3 *n* = 57

** All patients *n* = 147, Group 1 *n* = 45, Group 2 *n* = 52, Group 3 *n* = 50

*** Remaining patients who did not have a biopsy type indicated did not have a biopsy report available to review

IQR interquartile range. LTFU lost to follow up. US ultrasound

## Results

### Patient demographics and risk factors for head and neck squamous cell carcinoma patients

In 2018, 256 patients with HNC presented to MNH or ORCI (Supplementary Table 1). Among these patients, 176 had a pathologically confirmed diagnosis of HNSCC (Table 1). Among patients with HNSCC, the majority were males (67%, *n* = 118) and a large proportion of patients did not have relevant risk factors documented. Tobacco use was unknown for 27.8%(49), alcohol use was unknown for 23.9%(42), and HIV status was unknown for 72.2%(128).

### Clinical presentation and work up for head and neck squamous cell carcinoma patients

HNSCC patients presented with a median duration of symptoms of 24 weeks (IQR, 12–52 weeks). The most common imaging included that of the head and neck 85.2% (150 patients with at least one modality; 146 CT, 6 MRI, 6 neck ultrasound) and chest 84.1% (148 patients with at least one modality; 142 chest x-ray, 11 CT chest). A smaller proportion of patients underwent abdominal ultrasound 27.3%(48) (Table 1).

### Pathology subsite and staging for head and neck squamous cell carcinoma patients

The most common subsite was the oral cavity 31.8%(56) followed by larynx 18.8%(33) and hypopharynx 14.2% [25]. Most patients presented with late-stage disease (39.7%, 70, stage III and IV combined). A large portion had missing stage (48.3%, 85) (Table 1). Testing for human papillomavirus and its surrogate p16 immunohistochemistry were not performed.

### Treatment for head and neck squamous cell carcinoma patients

**Fig. 1:**
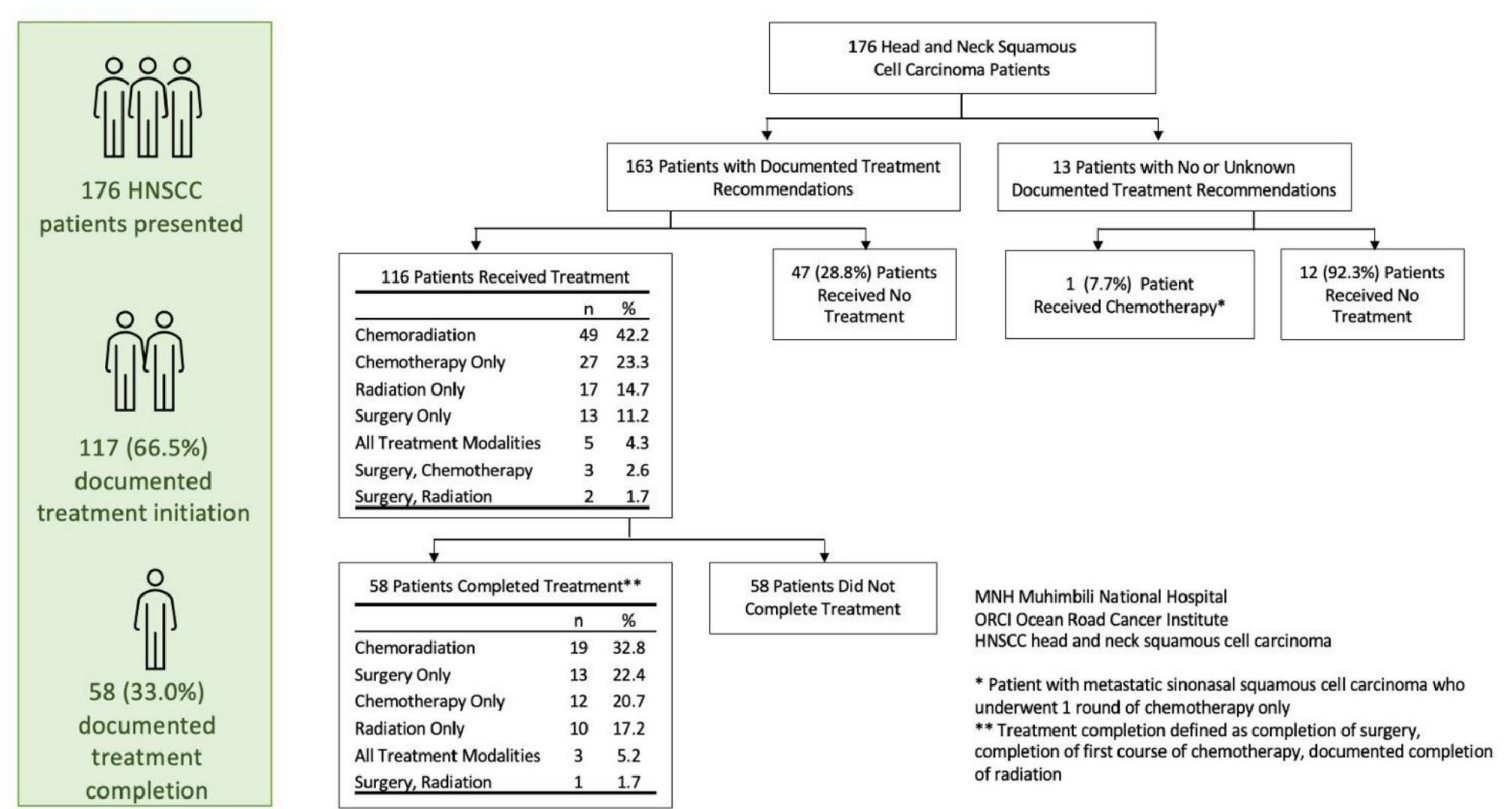
Treatment initiation and completion of head and neck squamous cell carcinoma patients

Among the 176 HNSCC patients following diagnosis, 33.5% (59) of patients had no treatment documented (group 1), 33.5%(59) had documentation of treatment initiation but not completion (group 2), and 33.0%(58) had documentation of treatment completion based on the modalities started (group 3, Table 1; Fig. 1). Among the 59 patients in group 1 who did not initiate treatment, reasons for not initiating treatment were clinical deterioration (*n* = 1), loss to follow up (*n* = 4), death (*n* = 2), or patient declined(*n* = 1). Among the 59 patients in group 2 who did not complete treatment, one patient was documented to be too deconditioned. There was no documented rationale for lack of treatment initiation or completion for remaining patients.

A significantly greater proportion of patients at ORCI (79.4%, 50/63) compared to MNH (59.5%, 67/113) initiated treatment (*p* = 0.007) whereas comparable proportions completed the planned treatment at both sites (MNH 31.9%(36/113), ORCI 34.9%(22/63), *p* = 0.679, Supplementary Table 2). The different proportions of patients who completed by modality is noted in Fig. 1.

### Univariate and multivariate analysis of predictors for treatment initiation and completion among head and neck squamous cell carcinoma patients

For treatment initiation (Table 2), late overall stage compared to early stage led to a significantly increased odds of initiating treatment (odds ratio, OR, 8.24, 95% CI 2.05–33.11, *p* = 0.003). No additional variables met the threshold of *p* < 0.10 for inclusion in the multivariate model.

**Table 2:** Univariate and multivariate analysis of predictors for treatment initiation among head and neck squamous cell carcinoma patients*

Predictor		Univariate Analysis		
		OR	95% CI	*p*
Age (years)		0.99	0.96–1.02	0.548
Location (residing outside of Coastal zone relative to in Coastal zone)		1.47	0.56–3.94	0.441
Late-stage relative to early stage		8.24	2.05–33.11	**0.003**
Subsite relative to oral cavity cancer				
	Larynx	0.69	0.20–2.43	0.566
	Hypopharynx	0.89	0.24–3.28	0.861
	Nasopharynx	1.21	0.27–5.40	0.801
Institution at presentation (ORCI relative to MNH)		1.31	0.29–6.01	0.726

*Patients with predictor variables missing were excluded from analysis

For the evaluation of predictors of treatment completion (Table 3) on univariate analysis, there was an increased odds of completing treatment in the case of late-stage disease relative to early stage disease (OR 7.41, 95% CI 0.90–60.99, *p* = 0.063) and decreased odds of completing treatment among patients with nasopharyngeal squamous cell cancers compared to oral cavity cancers (OR 0.14, 95% CI 0.02–1.34, *p* = 0.088) that met the threshold for inclusion into multivariate analysis. In multivariate analysis, no predictor variables remained significant.

**Table 3:** Univariate and multivariate analysis of predictors for treatment completion among head and neck squamous cell carcinoma patients*

Predictor		Univariate Analysis			Multivariate Analysis		
		OR	95% CI	*p*	OR	95% CI	*p*
Age (years)		1.00	0.97–1.04	0.331			
Location (residing outside of Coastal zone relative to in Coastal zone)		2.29	0.78–6.76	0.133			
Late-stage relative to early stage		7.41	0.90–60.99	**0.063**	8.43	0.92–77.31	0.059
Subsite relative to oral cavity cancer							
	Larynx	0.72	0.97–2.64	0.623			
	Hypopharynx	0.33	0.07–1.52	0.156			
	Nasopharynx	0.14	0.02–1.34	**0.088**	0.16	0.2–1.55	0.114
Institution at presentation (ORCI relative to MNH)		2.93	0.65–13.21	0.161			

*Patients with predictor variables missing were excluded from analysis

### Vital status

Among all patients with a median follow up of 5.6 months (IQR 1.64—12.5 months), 36.9%(65) were alive per last documented status in their medical records, 7.4% [13] were referred to palliative care, 21.6%(38) had died, and 34.1%(60) were lost to follow up (Supplementary Table 4).

## Discussion

This retrospective study of HNC patients who presented to the largest public, cancer referral hospitals in Tanzania noted that a third of patients did not initiate treatment and a third of patients did not complete treatment. Presentation with late-stage disease was associated with an increased odds of treatment initiation and completion. In our experience, patients with late-stage disease expressed greater intention to follow through with treatment when their symptoms and pain were more severe. Higher symptom burden serving as a motivation for treatment for patients and potentially providers therefore could underly the association between late-stage disease and treatment initiation and completion.

This study supplements the limited literature on treatment initiation and completion among HNC patients in Africa. The 33.5% of patients in this cohort who did not initiate treatment was higher compared to the 2.3% among nasopharyngeal cancer patients in Morrocco and 28% among laryngeal cancer patients in Ghana [15, 16]. Regarding treatment incompletion, this cohort had lower treatment incompletion compared to the 70% of treatment incompletion among laryngeal cancer patients in Ghana and the 40% treatment incompletion due to mortality alone among laryngeal cancer patients in Nigeria [16, 17]. Though the proportion of treatment incompletion among cancer patients is generally lower in high-income countries [18–20], certain populations such as HNC patients on government-sponsored health insurance in the United States have reported up to 40% of patients experiencing treatment interruptions or incompletion [21]. Incompletion of cancer care treatment is unsurprisingly associated with poorer survival [19, 22], and therefore serves as a critical metric to contextualize care and an opportunity for interventions.

These data reemphasize the need to better understand and intervene upon barriers to treatment for HNC patients in Africa. In qualitative studies from Tanzania, lack of knowledge, delayed presentation, and lack of financial resources were major barriers to care for patients [23–25]. On the health systems level, lack of education among healthcare providers and inefficient referrals to tertiary referral centers are common [23–25]. While studies have highlighted barriers to care for cancer patients overall, few studies pertain to HNC patients in sub-Saharan Africa [26–28]. Among these limited studies, the majority describe barriers to presentation and diagnoses but only one study identified barriers to treatment completion, which included chemotherapy shortages, dysfunctional radiotherapy machines, and inability of patients to afford transportation [27]. 

HNC is a treatable and curable cancer if diagnosed in a timely fashion and treated according to care standards; therefore, identifying modifiable barriers to care and treatment offers opportunities for interventions. Interventions to address delays to initiate treatment include education of patients and providers, expedited pathologic diagnoses, and consideration of HNC screening in resource-constrained health settings [29, 30]. To support treatment completion, patient navigation services in LMICs have modeled successful increase to treatment adherence [31]. Finally, financial support will be critical at all stages of care. These interventions are applicable to care at MNH and ORCI. Opportunities for improving treatment initiation and completion include patient education and social supports for transportation at the patient level. At the hospital level, there are opportunities to improve multidisciplinary care coordination and support from a patient care navigator. Finally, at a national level, the continued increase in cancer care workforce and infrastructure as well as developing systems for earlier disease detection are opportunities to improve access to HNC care.

While this study highlights the additional research needed to enhance treatment initiation and completion, these findings are limited by the retrospective nature of the study and limitations of available medical records. A large proportion of data was missing for critical clinical variables such as stage of disease. Additionally, the data was not linked to records at other treatment facilities and potentially incomplete. National cancer registries may help ameliorate this issue though are resource intensive to maintain particularly in resource-constrained health systems [32]. Furthermore, these data capture one time interval of HNC care. Given the concerted national efforts to improve cancer care since 2018 and implementation of Tanzania’s National Cancer Treatment Guidelines in 2021, these data may serve as a baseline for subsequent evaluations of HNC care delivery.

## Conclusion

This retrospective study of HNC patients in Tanzania highlights the need to develop interventions for addressing barriers to treatment initiation and completion along with improved reporting of treatment courses for patients to contextualize clinical outcomes.

## Electronic supplementary material

Below is the link to the electronic supplementary material.

Supplementary Material 1

## Data Availability

The data that support the findings of this study are available from the authors (MJX, AN) upon request. Permission and data transfer agreements may be needed under the approval of the Institutional Review Boards.
