# Supplementary material for: Treatment initiation and completion among head and neck squamous cell carcinoma patients in Tanzania
Source: BMC Res Notes. 2024 Dec 23;17:380. doi: 10.1186/s13104-024-07045-7 (PMC11668109; doi:10.1186/s13104-024-07045-7)
Supplement: Supplementary file 1 — Supplementary Material 1 [file 13104_2024_7045_MOESM1_ESM.docx]

Appendix: Assessment of Missingness of Data

Missingness at random was assessed for overall stage. This was done because there was the greatest proportion of missing data (>40%) compared to the other predictor variables included in the logistic regression, which were all <2%.

To test whether missingness was at random, first stage was coded as documented (1) vs missing (0). Then chi-square test was used to compare missing stage (stage_missingness) with treatment initiation (txnitiatedscc) and treatment completed (txcompletionscc) for head and neck squamous cell carcinoma patients.

Given that there was significance in comparing missingness of stage and treatment initiation, stage can not be assumed to be missing at random. To minimize biases, the logistic regression was therefore performed excluding patients with missing predictor variables.
